# The Non-Concordance of Self-Reported and Performance-Based Measures of Vestibular Dysfunction in Military and Civilian Populations Following TBI

**DOI:** 10.3390/jcm11112959

**Published:** 2022-05-24

**Authors:** Nicholas I. Wood, James Hentig, Madison Hager, Candace Hill-Pearson, Jamie N. Hershaw, Alicia R. Souvignier, Selena A. Bobula

**Affiliations:** 1 Evans Army Community Hospital, Fort Carson, CO 80913, USA; nicholas.i.wood2.ctr@mail.mil (N.I.W.); candace.a.pearson2.ctr@mail.mil (C.H.-P.); jamie.n.hershaw.ctr@mail.mil (J.N.H.); alicia.r.souvignier.mil@mail.mil (A.R.S.); selena.a.bobula.mil@mail.mil (S.A.B.); 2 Traumatic Brain Injury Center of Excellence, Fort Carson, CO 80913, USA; madison.g.hager.mil@mail.mil; 3 General Dynamics Information Technology, Falls Church, VA 22042, USA

**Keywords:** vestibular dysfunction, traumatic brain injury, TBI, military service members, activities-specific balance confidence, dizziness handicap inventory, CDP–SOT

## Abstract

As a predominately young, physically active, and generally healthy population, service members (SMs) with vestibular dysfunction (VD) following a TBI may not be accurately represented by the current civilian reference ranges on assessments of VD. This study enrolled SMs who were referred for vestibular rehabilitation following a mild/moderate TBI. The participants self-reported VD using the Activities-specific Balance Confidence (ABC) scale and the Dizziness Handicap Inventory (DHI) followed by evaluation of vestibular performance using computerized dynamic posturography sensory organizational test (CDP–SOT). Retrospective analysis of these outcomes comparing the study sample of SMs to the reported civilian samples revealed SMs self-reported lower VD with significantly higher balance confidence (ABC: 77.11 ± 14.61, *p* < 0.05) and lower dizziness (DHI: 37.75 ± 11.74, *p* < 0.05) than civilians. However, the SMs underperformed in performance-based evaluations compared to civilians with significantly lower CDP–SOT composite and ratio scores (COMP: 68.46 ± 13.46, *p* < 0.05; VIS: 81.36 ± 14.03, *p* < 0.01; VEST: 55.63 ± 22.28, *p* < 0.05; SOM: 90.46 ± 10.17, *p* < 0.05). Correlational analyses identified significant relationships between the ABC and CDP–SOT composite (r = 0.380, *p* < 0.01) and ratio scores (VIS: r = 0.266, *p* < 0.05; VEST: r = 0.352, *p* < 0.01). These results highlight the importance of recognizing and understanding nuances in assessing VD in SMs to ensure they have access to adequate care and rehabilitation prior to returning to duty.

## 1. Introduction

Traumatic brain injuries (TBI) have increasingly become a major public health concern in both civilian and military populations, where a high prevalence among military service members (SMs) is attributed to combat operations or training-related tasks [1,2]. More than 400,000 SMs experienced TBIs during the Global War on Terrorism, with the majority classified as mild TBIs (mTBI) [3]. Although many symptoms of mTBIs often subside, there are frequent reports of persistent sequelae following a TBI such as dizziness, imbalance, and vertigo due to deficits in the vestibular system, which integrates multisensory information and is vulnerable to TBIs. These symptoms can result in acute and/or chronic functional limitations that can affect return to duty and unit readiness [4,5,6,7].

Short- and long-term consequences following a TBI that result in vestibular dysfunction were previously studied using various performance-based measures for balance and self-reported symptom severity and functional limitations [7,8]. Performance-based balance measures have been developed to objectively capture symptom burden for TBIs; however, balance measurement tools such as Functional Gait Assessment and Computerized Dynamic Posturography may lack the sensitivity to capture instability in high-functioning populations such as military SMs or collegiate athletes [9,10,11]. These balance assessments commonly used in clinical practice have demonstrated mixed reports with SMs at times underperforming civilians, while others demonstrate a ceiling effect where researchers have notably distinguished that SMs with a TBI exhibited scores that fell within the normal range or outperformed the civilian reference ranges [9,10,11]. The prevalence of TBIs in SMs and the substantial portion of TBI patients reporting vestibular-related impairments [7,12,13] make it necessary to identify performance-based assessments that are sensitive to subtle vestibular deficits in high-performing populations.

Although performance-based outcomes are critical for quantitatively identifying deficits and tracking recovery, self-reported factors are important for developing treatment plans, qualifying complaints, and enhancing patient compliance [14,15,16]. Subjective self-report measures often include the Dizziness Handicap Inventory (DHI), the Rivermead Post-Concussion Questionnaire (RPQ), the Sport Concussion Assessment Tool (SCAT3), and the Post-Concussion Symptom Scale (PCSS), which have been shown to support complaints of imbalance [17,18,19,20,21]. However, inconsistent findings have been reported regarding the relationships between the patients’ perceived ability and actual performance on vestibular-related functional tasks [21,22,23]. These inconsistencies have been found in both civilian and military populations. Both Inness [21] as well as Moore et al. [24] reported a dissociation between subjective and objective balance measures in civilians, whereas Herbert [7] demonstrated significant relationships between subjective self-reported dizziness using the Dizziness Handicap Index (DHI) and objective balance measured by the computerized dynamic posturography sensory organization test (CDP–SOT) in Operation Iraqi Freedom (OIF)/Operation Enduring Freedom (OEF) veterans.

Discerning which measures are appropriate in military populations for detecting effects of TBIs on vestibular performance and symptom presentation can support the clinicians’ ability to better assess, diagnose, treat, and inform return-to-duty decisions for SMs. This study intends to evaluate the relationships between self-reported and performance-based assessments of vestibular function in a sample of SMs seeking treatment for vestibular impairment following a TBI and compare these measures between SM and comparable civilian-reported outcomes.

## 2. Materials and Methods

### 2.1. Design and Participants

This study utilized a single-site randomized enrollment of active-duty SMs between 18 and 49 years of age who were referred for vestibular rehabilitation with a history of mild or moderate TBI. Study-trained providers identified and referred interested patients to research staff who verified eligibility and enrolled participants. All the enrolled participants reported a mild or moderate TBI in accordance with the ACRM criteria [25] and confirmed by the Ohio State University TBI Identification (OSU TBI ID) tool [26] that occurred between 4 weeks to 5 years prior to study enrollment. Only individuals who self-reported dizziness symptoms as indicated with a score between 16 and 64 on the Dizziness Handicap Inventory (DHI) were included in the study. The participants had pre-intervention (baseline) and post-intervention assessments before and after vestibular rehabilitation treatment, in which intervention outcomes were previously assessed and compared by Loftin [27] and Vander Vegt [28]. Only baseline assessments of the same sample were used in our analysis. Of the 62 individuals enrolled into this study, six participants were excluded from analyses for incomplete baseline data or outcome measures that exceeded three standard deviations. The participant demographics for the final sample that was included in our analyses are found below. The Institutional Review Board of the Regional Health Command—Atlantic approved the protocol for this study. All the participants provided signed informed consent prior to their participation and after all the procedures were explained.

### 2.2. Procedures

Pre-intervention evaluation consisted of the collection of basic demographics, medical history information, confirmation of a mild or moderate TBI using the OSU TBI ID, and completion of an assessment battery that included a variety of self-reported and performance-based metrics to capture the spectrum of the vestibular symptom burden associated with TBIs. Self-reported measures included the Dizziness Handicap Inventory (DHI) and the Activities-specific Balance Confidence scale (ABC); the primary performance-based measure used was the computerized dynamic posturography sensory organization test (CDP–SOT), which provides a composite (COMP) score and visual (VIS), vestibular (VEST), and somatosensory (SOM) ratio scores. Additional performance-based measures assessed as part of the larger study included the head shake SOT, the dynamic visual acuity test, and the neuro-otologic test system (NOTC). However, results from these assessments were not included in the current analysis due to limited availability of comparison studies in civilian populations.

A total of 14 comparative peer-reviewed studies in civilians (Table 1) were identified for statistical comparison with our active-duty military sample. These studies were identified by searching the following keywords: “vestibular therapy,” “TBI/mTBI,” “concussion,” “military TBI,” etc. We selected studies with similar metrics and varying civilian patient populations that reported summary data for the DHI, the ABC, or the CDP–SOT.

### 2.3. Activities-Specific Balance Confidence Scale (ABC)

The ABC scale is used to assess confidence while performing various balance-dependent tasks. This 16-item measure has the respondents rank their ability to perform common daily activities (e.g., walking, reaching, using an escalator, and bending) from 0% (no confidence) to 100% (complete confidence). This survey produces one overall confidence number computing an average confidence rating over all the items. The ABC scale is valid, has been recognized to have high internal consistency, and has demonstrated the sensitivity to discriminate between individuals with and without falls in patients with vestibular impairments (alpha = 0.91) [29].

### 2.4. Dizziness Handicap Inventory (DHI)

The DHI is a 25-item self-reported questionnaire to quantify the perceived impact of dizziness-related deficits. The items are answered using three response levels: “no” is scored as 0 points, “sometimes” is scored as 2 points, and “yes” is scored as 4 points, with the maximum score of 100. A higher score indicates that the respondent perceives a higher impairment. The DHI is valid and has demonstrated high internal consistency for quantifying self-perceived vestibular handicap (alpha = 0.89) [14].

### 2.5. Computerized Dynamic Posturography Sensory Organization Test (CDP–SOT)

The CDP–SOT was assessed using the NeuroCom^®^ Balance Manager SMART EquiTest^®^ clinical research system (CRS) (Natus Medical Inc., Pleasanton, CA, USA), which consists of a fixed visual surround as the participants stand with standardized foot placement on a moveable dual force plate. The patients are harnessed as they perform three trials, each of six conditions: (1) eyes open, no sway; (2) eyes closed, no sway; (3) eyes open, visual/surround sway; (4) eyes open, support surface sway; (5) eyes closed, support surface sway; and (6) eyes open, support surface and visual/surround sway. Comparisons between these six conditions yield four center-of-pressure postural control scores. The CDP–SOT equilibrium composite score represents overall balance with a higher number indicating better balance; it is the weighted average of all the six conditions. A score of 0 means the patient fell and a value of 100 indicates perfect stability. The visual ratio represents the input of the visual system for maintaining balance and stability; it is computed by dividing the average performance in condition 4 by the average in condition 1. The somatosensory ratio represents a sensation produced by mechanical forces (e.g., proprioception, pressure, and touch), determined by dividing the average performance in condition 2 by the average performance in condition 1. The vestibular ratio represents the stability when visual and somatosensory inputs are removed; it is determined by dividing the average performance in condition 5 by the average performance in condition 1. Ratio scores are reported as whole numbers. The CDP–SOT shows a moderate level of reliability [30].

### 2.6. Statistical Analysis

For comparative studies that reported the means, standard deviations, and sample size, we used two-tailed independent-samples *t*-tests, and for comparisons to studies with only published means and sample sizes, we used one-sample *t*-tests for group comparison to our sample. Cohen’s d for effect size of independent-samples *t*-tests were calculated with pooled variance, while Cohen’s d for effect size of one-sample *t*-tests used the standard deviation of the military sample. To examine the relationships between the self-reported measures (DHI and ABC) and the performance-based metrics (CDP–SOT composite and ratio scores), we computed Pearson’s correlation coefficients. All data analyses were performed using SPSS version 27.0 (SPSS Inc., Chicago, IL, USA) using two-tailed tests with an α value set at 0.05.

## 3. Results

### 3.1. Demographic Characteristics

Our military sample included 56 active-duty SMs with an average age of 32.52 years (SD = 8.17), who were predominately male (83.83%) and identified as White (62.5%). The sample had an average of 4.19 lifetime TBIs (SD = 3.7), with 46 SMs (82.14%) sustaining a blunt-force TBI as their most recent mechanism of injury (MOI, 17.85% blast-force TBI) and an average time since injury (TSI) of 13.63 months (SD = 16.17) since their most recent TBI (Table 1). In contrast, seven comparable civilian studies had a mean age significantly higher than that of our military cohort (civilian studies: a–d, j, *p* < 0.01, h, n, *p* < 0.05), and five studies’ samples were significantly younger (e, g, k–m, *p* < 0.01, Table 1). Additionally, all the civilian studies that reported gender included a higher percentage of women (a, b, d, f, h–k, m; the percentage of men ranged from 33.33% to 69.44%, Table 1), and the TSI was significantly different between our military sample and most comparable civilian studies that reported TSI (g, h, j–m, *p* < 0.01, Table 1). Full demographic and clinical characteristics, including sample means, standard deviations, and ranges for all subjective and objective measures for our military sample and comparable civilian studies of adults with post-concussion vestibular symptoms are summarized in Table 1.

**Table 1:** Demographic characteristics of the study participants and comparable civilian studies.

	Military Sample (*n* = 56)	a (*n* = 214)	b (*n* = 92)	c (*n* = 44)	d (*n* = 10)	e ABC (*n* = 58) DHI (*n* = 59)	f (*n* = 8)	g ABC (*n* = 129) DHI (*n* = 127)	h (*n* = 10)	i (*n* = 6)	j (*n* = 100)	k (*n* = 108)	l (*n* = 62)	m (*n* = 12)	n (*n* = 48)
**Authors**. **Reference**	–	Vereeck et al., 2007 [22]	Tamber et al., 2009 [31]	Findling et al., 2011 [32]	Basford et al., 2003 [33]	Alsalaheen et al., 2016 [34]	Moore et al., 2016 [24]	Dunlap et al., 2020 [35]	Kaufman et al., 2006 [17]	Adams & Moore, 2017 [36]	Whitney et al., 2006 [37]	Register-Mihalik et al., 2008 [38]	Sosnoff et al., 2011 [39]	McDevitt et al., 2016 [40]	Row et al., 2019 [41]
**Age-**(years), mean SD	32.52 ±8.17	53.9 ** ±13.5	47.2 ** ±11.46	38.4 ** –	40.9 ** ±11.3	15 ** ±1.8	31 –	20 ** ±7	41 * ±11	39.33 ±13.7	59 ** ±17	18.83 ** ±1.27	20.04 ** ±1.47	20.5 ** ±1.8	47.49 * ±16.12
**Gender-**Males, n (%)	47 (83.93%)	110 (51.4%)	28 (30.43%)	–	6 (60%)	20 (33.89%)	4 (50%)	–	6 (60%)	2 (33.33%)	38 (38%)	75 (69.44%)	–	7 (58.3%)	–
**Race-**White, n (%)	35 (62.5%)	–	–	–	–	–	–	–	–	–	–	–	–	–	–
**# of TBI-**mean SD	4.19 ±3.7	–	–	–	–	–	–	–	–	1.67 ** ±1.21	–	–	1.24 ** ±0.61	2.6 ±2.9	–
**MOI-**Blunt, n (%)	46 (82.14%)	–	–	–	–	–	–	–	–	–	–	–	–	–	–
**TSI-**(months), mean SD	13.63 ±16.17	–	–	–	–	–	10.68 –	7.07 ** ±7.92	33.60 ** –	19.84 ±10.66	36 ** ±60	0.05 ** ±0.03	44.3 ** –	0.92 ** –	12.52 ±13.70

ABC = Activities-specific Balance Confidence scale, DHI = Dizziness Handicap Inventory, MOI = mechanism of injury, SD = standard deviation, TBI = traumatic brain injury, TSI = time since injury, * *p* < 0.05, ** *p* < 0.01; bold values represent significant findings.

### 3.2. Service Members Self-Report Fewer Vestibular Deficits Following TBIs

We compared the total ABC score in our military sample to five civilian studies (e–g, i, j) that also utilized the ABC scale. In our military sample, we observed an average total ABC score of 77.11 (SD = 14.61). In contrast, three civilian studies that collectively spanned adolescents (e), age- and TSI-matched (f), and late adulthood (j) reported scores that were significantly lower compared to the self-reported perceived balance ability in our military sample (civilian studies: e, *p* < 0.05, d = 0.47; f, *p* < 0.05, d = 0.35; j, *p* < 0.01, d = 0.86; Table 2), while one study reported significantly higher scores (civilian study: g, *p* < 0.01, d = 1.51; Table 2). We then compared the perceived impact of dizziness-related deficits in our military sample to nine civilian studies that included the DHI (a–i). Our military sample reported an average DHI score of 37.75 (SD = 11.74), while five civilian studies reported significantly different total DHI scores (*p* < 0.05). Specifically, our military sample self-reported a significantly lower dizziness-related impairment compared to four civilian studies (c, *p* < 0.01, d = 2.12; e, *p* < 0.05, d = 0.38; f, *p* < 0.01, d = 1.29; i, *p* < 0.05, d = 1.52; Table 2). However, one civilian study (g) did report a significantly lower patient dizziness-related impairment compared to our military sample (civilian study: g, *p* < 0.01, d = 1.99; Table 2).

### 3.3. Service Members Experience More Performance-Based Vestibular Impairment Following a TBI

We next compared vestibular impairment indexed by the CDP–SOT performance, including composite and visual, vestibular, and somatosensory ratio scores, between our military sample and comparative civilian studies, which were limited to three studies of collegiate athletes (k–m) and one study in the general population (n). We observed an average CDP–SOT composite score of 68.46 (SD = 13.46) in our military sample, while two athletic studies reported significantly higher total composite scores compared to our military sample (l, *p* < 0.01, d = 2.42; m, *p* < 0.05, d = 0.68; Table 3). However, when we examined the CDP–SOT scores by visual, vestibular, and somatosensory ratio scores of our military sample and the four comparable civilian studies (k–n), we observed significant differences in at least two of the three ratios in all the four studies (Table 3). Though our military sample reported lower visual ratio scores (81.36, SD = 14.03) than all the four comparable civilian studies (k–n), visual ratio scores were only significantly different than in one athletic study (l, *p* < 0.01, d = 1.57) and civilian study n (*p* < 0.01, d = 0.56; Table 3). In contrast, our military sample showed significantly lower vestibular ratio scores (55.63, SD = 22.28) than all the three civilian athletic studies (k, *p* < 0.05, d = 0.33; l, *p* < 0.01, d = 2.19; m, *p* < 0.01, d = 0.85; Table 3) and lower somatosensory ratio scores (90.46, SD = 10.17) than all the four civilian studies, athletic or otherwise (k, *p* < 0.01, d = 0.66; l, *p* < 0.01, d = 1.08; m, *p* < 0.05, d = 0.56; n, *p* < 0.05, d = 0.42; Table 3).

### 3.4. Interrelationship of Self-Reported and Performance-Based Vestibular Measures in Military Service Members

Finally, Pearson’s correlations were calculated to examine relationships between self-reported and objective performance-based measures in our military cohort. Within our military sample, we identified significant positive relationships between the patients’ perceived balance ability and their balance performance scores (Table 4). Specifically, significant positive associations were identified between the ABC and the CDP–SOT total composite scores (*p* < 0.01), visual ratio scores (*p* < 0.05), and vestibular ratio scores (*p* < 0.01, Table 4). We did not observe any significant associations between the DHI and any of the CDP–SOT measures.

## 4. Discussion

The strict requirements for military service results in an SM population that is predominately young, healthy, physically active, and may not be accurately represented by the general population. Here, we evaluated vestibular-related impairment measures in an active-duty military sample with a history of TBI and then compared the baseline evaluations of self-reported and vestibular performance-based measures to previous studies with civilian samples. The goals of this retrospective analysis were twofold: (i) to determine the relationship between active-duty SMs with mild to moderate TBIs and the broader civilian population with similar injuries and (ii) to assess the correlation between the self-reported and performance-based measures of symptom burden in the military TBI cohort.

Although TBIs are of higher prevalence in young adolescent and geriatric general populations [42], the paucity of research towards 24–65-years-old individuals predominately excludes SMs who face an increased occupational risk of TBIs. Similarly, TBI-related vestibular studies in civilians have largely centered around either adolescents/young adults with athletic injuries or older adults with age-related vestibular impairments and can have a wide range of the TSI. We observed a similar trend among the 14 comparable civilian studies in our analysis wherein the average age of civilian participants was significantly younger or older than in our military sample and sample sizes were limited in the two age-matched studies (f and j).

Comparing the ABC scores, our military sample showed a notable difference from comparable civilian populations in four of the five studies (e, f, g, j) that used the measure. The military population generally showed greater confidence in self-reported measures of balance than civilian studies in the general population with significant age differences. This is potentially due to the demographic nature of the military population as young and generally healthy individuals. Between the military sample and an age-matched civilian study (i), there was no detectable difference in confidence, while a young adult study (g) reported much higher confidence levels. Proprioception performance has been shown to be age-related, with peak acuity in young adults followed closely by middle-aged individuals [43,44]; it may explain an increased confidence level in the military sample.

The self-reported symptom burden varied with comparison to civilian studies with our military sample reporting similar DHI scores as half of the civilian samples, and notably lower than the other half of civilian studies. With the exception of one civilian study (g) reporting a significantly lower DHI score, most reports of the DHI, both military and civilian, fell into the moderate disability range, with two civilian studies (c and i) breaching severe scores [14]. Duration of symptoms, more so than age, has been associated with increased DHI scores [45], and not having the TSI for all the comparison studies limits interpretation of these data. Additionally, the report of lower symptom burden may also be a result of military culture as self-reporting injuries has inconsistent reliability in studies of SM populations [46,47].

When we compared vestibular performance-based CDP–SOT composite and respective ratio scores, poorer performance was found in our military sample in nearly every study comparison. These comparisons included both comparable physically active collegiate athletes as well as an older general civilian population. Collectively, this suggests that the SMs experienced greater performance-based vestibular-related deficits following a mild or moderate TBI, though they self-reported fewer deficits than civilians. It is possible that SMs, while more objectively impacted by their injury, report a lesser degree of subjective symptom burden as a result of military culture. These findings reinforce the importance of a comprehensive approach to TBI diagnosis, treatment, and rehabilitation in this population.

The second objective of this investigation assessed relationships between self-reported and performance-based measures of symptom burden within the military population. The outcome data suggest that the ABC is a more useful indicator of objective symptom burden among active-duty SMs. The data suggested limited ability for the DHI to predict or approximate the vestibular performance impairment in our military TBI population. This result could be a byproduct of the nature of the DHI and ABC surveys as the ABC has great specificity with regard to the specific balance tasks measured in the CDP–SOT. While the DHI includes some specific balance and mobility questions, it also encompasses broader symptom domains, including psychosocial factors. Although the ABC appears to better approximate the vestibular performance in military patients with a TBI, and eliminating the DHI may streamline the standard of care, clinicians may choose to retain the DHI to address the many facets of brain injury and ensure active-duty SMs receive adequate care.

Although the current study adds to the shortfall of research in individuals aged 24–65 years with a history of TBI and addresses specific gaps related to military health, this study is not without limitations. This study was limited in scope due to its nature as a retrospective analysis of data collected for a clinical trial that measured pre-/post-intervention differences on a battery of additional objective metrics. Our sample size consisted of the data available from the initial pre-intervention assessment, and the aims of this analysis may have been better served with a more robust sample size.

Demographically, limited comparable studies were found, consisting of similarly aged civilians with head injuries that employed the same self-reported and performance-based measures. These findings should be replicated with a comparable group to account for age-, TSI-, and sex-related differences following a TBI. Additionally, unique to SMs, Roberts et al. [48] suggested within-group variability on occupational characteristics, such as differences in occupational specialties and training experiences, may cause variability in the objective performance.

Other limitations in the current study are related to confines of which types of measures were examined in the civilian TBI/vestibular literature and the measures themselves. There is a paucity of work that includes both self-reported (ABC, DHI) and performance-based (SOT) measures collectively. Of our 14 comparable civilian studies, none examined both self-reported and performance-based measures, and only four studies looked at both the ABC and the DHI (e–g, i). Comparisons of our military cohort to literature where both subjective and objective measures were conducted in the same civilian population would be ideal but not possible given the body of previous studies. However, as to not severely limit our analyses, we chose to include a wide range of civilian studies that included either self-reported or performance-based measures. Furthermore, the current study only made statistical comparisons to two self-reported measures (ABC, DHI) and one performance-based measure (CDP–SOT). Although additional objective measures, particularly related to the vestibular ocular reflex, would provide a greater insight into TBI-induced vestibular-related decreased performance, the measures conducted in this study, the ABC, the DHI, and the CDP–SOT are validated and routinely used instruments that have clinical utility. We believe that the results of our study using these outcomes are meaningful and can inform clinicians about differences between military and civilian patients.

Lastly, this study did not have an ability to stratify individuals based on the number of TBIs sustained nor mechanism of injury, which may provide valuable insights. Future examinations focused on comparisons between military and civilian populations should strongly consider the mechanism of TBI. Unlike most of the civilian population, SMs are particularly susceptible to low-level primary blasts and have high rates of vestibular deficits following blast TBIs [49].

## 5. Conclusions

TBIs have been and will remain a significant problem for active-duty SM. While there are existing studies that examine civilian and military populations with TBIs, there are few studies that directly compare self-reported and performance-based measures of vestibular impairment within and between these two populations. Overall, this study found differences between a military sample and typical civilian TBI patients, both demographically and in terms of symptom burden and overall performance on vestibular activities. This paper intended to highlight the importance of recognizing and understanding these differences to ensure SMs have access to adequate care and rehabilitation prior to returning to duty. Future studies are necessary to elucidate the best assessment and treatment options for SMs suffering from vestibular deficits following a TBI based on population-specific factors and an understanding of military medicine and culture.

## Figures and Tables

**Table 2:** Comparison of the self-reported vestibular symptom burden following a TBI.

	Military Sample (n = 56)	a (n = 214)	b (n = 92)	c (n = 44)	d (n = 10)	e ABC (n = 58) DHI (n = 59)	f (n = 8)	g ABC (n = 129) DHI (n = 127)	h (n = 10)	i (n = 6)	j (n = 100)
**ABC-**mean SD	77.11 ±14.61	– –	– –	– –	– –	67 * ±27	72 * –	96 ** 10	– –	76.8 ±13.2	60 * ±24
**DHI-**mean SD Range	37.75 ±11.74 [16–62]	35.1 ±25 [0–96]	39.91 ±18.95 [4–86]	62.7 ** – [28–94]	32.2 ±23 [4–68]	44 * ±20 –	53 ** – [30–96]	12 ** ±14 –	32 ±23 [4–68]	64.1 * ±21.5 [30–96]	– – –

ABC = Activities-specific Balance Confidence scale, DHI = Dizziness Handicap Inventory, SD = standard deviation, * *p* < 0.05, ** *p* < 0.01; bold values represent significant findings.

**Table 3:** Comparison of the performance-based vestibular function by the CDP–SOT following a TBI.

	Military Sample (*n* = 56)	k (*n* = 108)	l (*n* = 62)	m (*n* = 12)	n (*n* = 48)
**CDP–SOT**					
**COMP**-mean SD	68.46 ±13.46	72.67 ±14.23	91.6 ** ±1.5	76.1 * ±8.5	72 ±12
**VIS**-mean SD	81.36 ±14.03	83 ±18	97 ** ±1.4	84 ±10	88 ** ±9
**VEST**-mean SD	55.63 ±22.28	63 * ±23	90.7 ** ±3.8	69 ** ±1	54 ±26
**SOM**-mean SD	90.46 ±10.17	95 ** ±6	98.3 ** ±1.1	95 * ±5	94 * ±6

CDP–SOT = computerized dynamic posturography sensory organization test, COMP = composite, SD = standard deviation, SOM = somatosensory subscore, VIS = visual subscore, VEST = vestibular subscore,* *p* < 0.05, ** *p* < 0.01; bold values represent significant findings.

**Table 4:** Pearson’s correlation coefficients of self-reported and performance-based measures.

	ABC		DHI	
	*r*	*p*	*r*	*p*
**COMP**	**0.380**	**0.004**	−0.239	0.076
**VIS**	**0.266**	**0.048**	−0.05	0.713
**VEST**	**0.352**	**0.008**	−0.23	0.089
**SOM**	0.220	0.103	−0.232	0.086

ABC = Activities-specific Balance Confidence scale, CDP–SOT = computerized dynamic posturography sensory organization test, COMP = composite, DHI = Dizziness Handicap Inventory, SD = standard deviation, SOM = somatosensory subscore, VIS = visual subscore, VEST = vestibular subscore; bold values represent significant findings.

## Data Availability

The anonymized clinical data that support the findings of this study are available upon reasonable request from any qualified investigator to the corresponding author.

## Disclaimer

The views expressed in this manuscript are those of the authors and do not necessarily represent the official policy or position of the Defense Health Agency, the Department of Defense, or any other US government agency. This work was prepared under contract No. HT0014-21-C-0012 with the DHA Contracting Office (CO NCR) HT0014 and, therefore, is defined as a US Government work under Title 17, USC § 101. Per Title 17, USC § 105, copyright protection is not available for any work of the US Government. For more information, please contact dha.TBICOEinfo@mail.mil. UNCLASSIFIED.
